# Small Deletions of *SATB2* Cause Some of the Clinical Features of the 2q33.1 Microdeletion Syndrome

**DOI:** 10.1371/journal.pone.0006568

**Published:** 2009-08-10

**Authors:** Jill A. Rosenfeld, Blake C. Ballif, Ann Lucas, Edward J. Spence, Cynthia Powell, Arthur S. Aylsworth, Beth A. Torchia, Lisa G. Shaffer

**Affiliations:** 1 Signature Genomic Laboratories, LLC, Spokane, Washington, United States of America; 2 Carolinas Medical Center, Clinical Genetics Center, Charlotte, North Carolina, United States of America; 3 Departments of Pediatrics and Genetics, University of North Carolina-Chapel Hill, Chapel Hill, North Carolina, United States of America; University of Missouri-Kansas City, United States of America

## Abstract

Recurrent deletions of 2q32q33 have recently been reported as a new microdeletion syndrome. Clinical features of this syndrome include severe mental retardation, growth retardation, dysmorphic features, thin and sparse hair, feeding difficulties and cleft or high palate. The commonly deleted region contains at least seven genes. Haploinsufficiency of one of these genes, *SATB2*, a DNA-binding protein that regulates gene expression, has been implicated as causative in the cleft or high palate of individuals with 2q32q33 microdeletion syndrome. In this study we describe three individuals with smaller microdeletions of this region, within 2q33.1. The deletions ranged in size from 173.1 kb to 185.2 kb and spanned part of *SATB2*. Review of clinical records showed similar clinical features among these individuals, including severe developmental delay and tooth abnormalities. Two of the individuals had behavioral problems. Only one of the subjects presented here had a cleft palate, suggesting reduced penetrance for this feature. Our results suggest that deletion of *SATB2* is responsible for several of the clinical features associated with 2q32q33 microdeletion syndrome.

## Introduction

Special AT-rich sequence binding protein 2 (SATB2; OMIM 608148) is a DNA-binding protein that regulates gene expression through chromatin modification and interaction with other proteins and plays roles in mammalian development [1]–[4]. In humans haploinsufficiency has been implicated in isolated cleft palate (CPI, OMIM 119540) [5], [6]. A single case of a *de novo* nonsense mutation of *SATB2* has been described in an individual with cleft palate, osteoporosis, profound mental retardation, epilepsy, a jovial personality, and craniofacial dysmorphism including gum hyperplasia, mandibular hypoplasia, and anterior-pointing incisors [7]. Three individuals with apparently balanced translocations with a breakpoint within *SATB2* or in a highly conserved region 3′ of the gene have also been reported [5], [8]. Two of these individuals had cleft palate, mild learning disabilities with speech delay and strikingly similar dysmorphic features with a prominent nasal bridge, underhanging columella, and a small mouth with a distinctive upper lip [6]. The third individual was diagnosed with Toriello-Carey syndrome because of the presence of Robin sequence, agenesis of the corpus callosum, dysmorphic features, and global developmental delay [8]. A fourth case of a balanced translocation predicted to disrupt the *SATB2* gene has been reported in an individual with autism spectrum disorder and developmental dyspraxia [9]. Mouse studies have shown a role for Satb2 beyond the palatal shelves, with roles in B-cell differentiation [2], osteoblast differentiation [4], establishment of neural corticocortical connections across the corpus callosum [3], and in the developing jaw and incisors [4], [10]. Because *SATB2* is highly conserved between species (99.6% between humans and mice), a deletion of this gene in humans is predicted to affect multiple systems.

Recently, Van Buggenhout et al. [11] used a 1-Mb bacterial artificial chromosome (BAC) microarray and characterized deletions of 2q32.2q33.3 in four individuals with severe mental retardation and growth retardation, facial dysmorphism, thin and sparse hair, micrognathia, cleft or high palate, persistent feeding difficulties, inguinal hernia, and broad-based gait. Three of these individuals had a distinctive behavioral phenotype with hyperactivity, motor restlessness, chaotic behavior, and happy personality with periods of aggression and anxiety. The authors suggested that, based on the common clinical features in these individuals and several previously reported individuals with cytogenetically visible deletions of 2q32q33 [12]–[27], microdeletions of 2q32q33 constitute a distinct syndrome (OMIM 612313). An additional case of this microdeletion has been reported in a male patient with a similar phenotype, including severe mental retardation, growth retardation, dysmorphic features, high palate, hyperactive and aggressive behavior, and hypotonia in infancy that changed to hypertonia [28]. Deletion of *SATB2* has been suggested to be responsible for the cleft or high palates in these individuals [11].

We report the clinical and molecular characterization of three individuals with microdeletions within 2q33.1 spanning part of the *SATB2* gene. From our review of the published literature these appear to be the smallest deletions associated with the 2q32q33 microdeletion syndrome reported to date and suggest haploinsufficiency of *SATB2* may be responsible for some of the clinical features associated with the syndrome.

## Results

**Figure 1:**
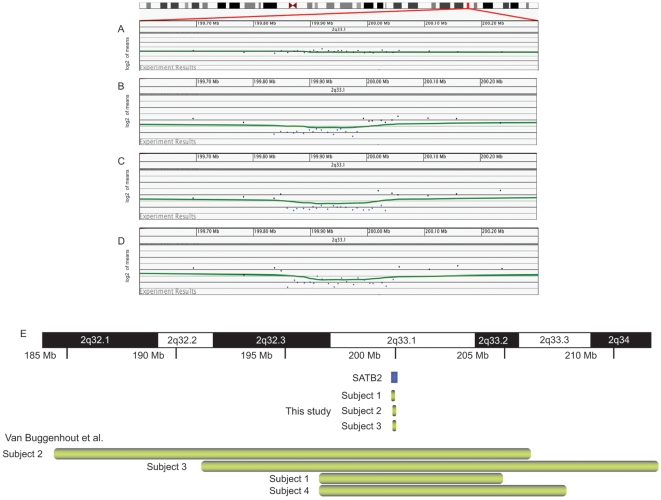
Analysis of individuals with microdeletions of 2q33.1. (A–D) Oligonucleotide microarray profiles for (A) normal chromosome 2, (B) a single-copy loss of 183.6 kb at 2q33.1 in subject 1, (C) a single-copy loss 173.1 kb at 2q33.1 in subject 2, and (D) a single-copy loss of 185.2 kb at 2q33.1 in subject 3. For the microarray plots, clones are ordered on the x axis according to physical mapping positions with proximal 2q33.1 to the left and distal 2q33.1 to the right. (E) Summary of the deletion sizes in individuals with microdeletions encompassing 2q33.1. Green bars indicate the approximate deletion sizes in individuals in the Van Buggenhout et al. [11] study (upper portion of diagram) and the current study (bottom of diagram). The *SATB2* gene is indicated by a red box.

We identified three individuals with microdeletion of 2q33.1 among our patient population. We characterized the deletions in more detail using a high-density 105K oligonucleotide microarray. Microarray analysis identified single-copy losses of the region containing *SATB2* in each of the individuals (Figure 1). A 183.6 kb deletion spanning all but the 5′ end of the *SATB2* gene at 2q33.1 (chr2:199,837,205–200,020,800) was found in subject 1; a 173.1 kb deletion at 2q33.1 within the *SATB2* gene (chr2:199,860,227–200,033,309) was found in subject 2; and a 185.2 kb deletion at 2q33.1 encompassing all but the 3′ end of *SATB2* (chr2:199,860,027–200,045,201) was found in subject 3. The only gene contained in each of the three deletions was *SATB2*. No other clinically significant gains or losses were detected in these individuals. A search of the Database of Genomic Variants (projects.tcag.ca/variation/) showed seven normal individuals have been reported with small deletions in *SATB2*; however, each of the deletions was entirely within an intron of the gene.

FISH using BAC probe RP11-404F23 to the *SATB2* locus confirmed a deletion in all subjects. Parental specimens were available only for the mothers of subjects 1 and 2, and neither mother carried the deletion of 2q33.1. Biological parents of subject 3 were unavailable for testing.

**Figure 2:**
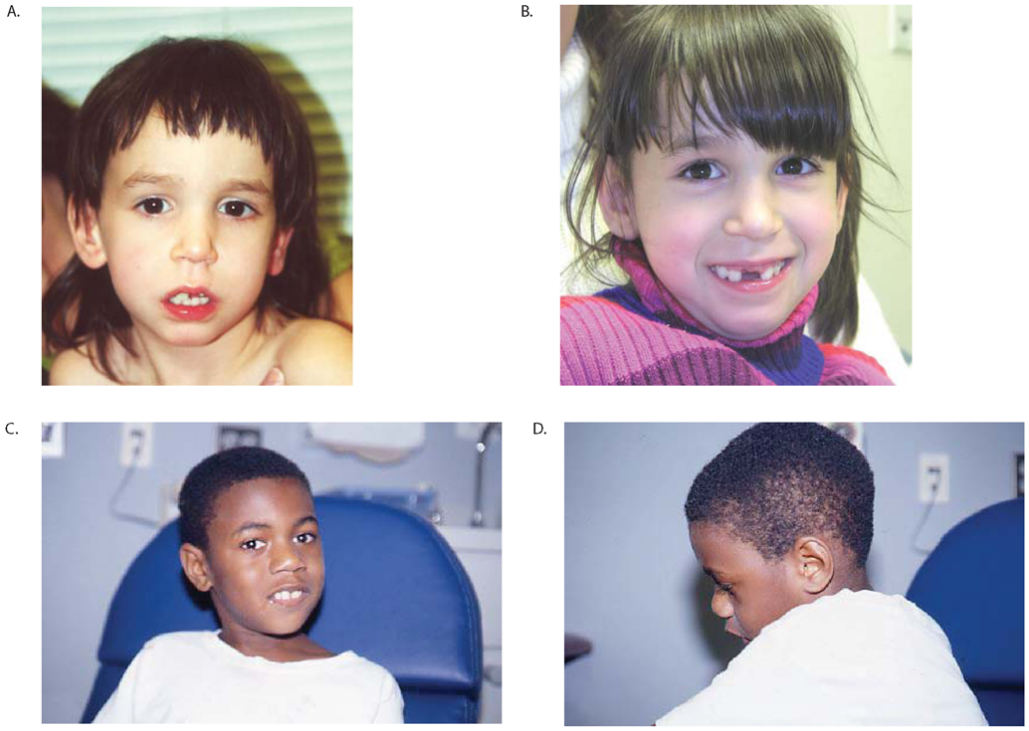
(A) Facial image of subject 1 at age 2 years, 9 months. (B) Facial image of subject 1 at age 9 years, 8 months. Subject is in mixed dentition and is missing her maxillary central incisors. (C) Front and (D) profile facial images of subject 2 at age 10 years. Note the broad nasal midsection, small mandible, and macrocephaly.

Clinical information was available for subjects 1–3 (Table 1). Subject 1 is a 9-year 8-month old female with severe mental retardation, dysmorphic features, and behavior problems. Family history was negative for any individuals with similar developmental delays. The subject was born to a 30-year-old mother via a somewhat difficult vaginal delivery following a pregnancy complicated by gestational diabetes. Fetal activity was normal. Exposures were denied. There was perinatal distress with difficulty in delivering the head. Labor was induced. Birth weight was 8 pounds 9 ounces. She had a fractured clavicle and some transient tachypnea and was discharged home at 7 days. Developmental delays were noted in early childhood, and intelligence testing placed her IQ below 50. She had delayed primary dentition, with eruption of her first tooth at age 2. At age 9 years 8 months she has 20 words, four to five signs, and one two-word phrase. Head MRI at age 2 was normal, and head CT at age 7 was also unremarkable. She has a heart murmur with no known further workup. Dysmorphic features include frontal bossing, a long and narrow face with a prominent maxillary arch, a class II malocclusion with relative micrognathia, slightly large ears, a smooth upper lip, and a smooth philtrum (Figure 2A–B). The subject was uncooperative with direct examination of her palate, but her history is not consistent with any palatal clefting. At age 9 years 8 months height is at the 25^th^–50^th^ percentile, weight is at the 50^th^–75^th^ percentile, and head circumference is at the 75^th^ percentile. She has normal dentition. The subject is very social, repeats actions multiple times, frequently washes her hands, likes to have her hair washed, has touch avoidance, has good eye contact, is somewhat photophobic, and has nodules on the dorsal surfaces of her fifth fingers bilaterally consistent with the effects of chewing on her hands.

**Table 1:** Summary of clinical features in individuals with deletions encompassing 2q33.1 in this and previous studies.

	Individuals in this report	2q32q33 microdeletion syndrome cases [11], [28]	Individuals with reported cytogenetically detected deletions encompassing 2q33.1^a^	Individuals with *SATB2* disruption caused by balanced translocation [6], [8]
**Neurological**				
Severe developmental delay/mental retardation	3/3	5/5	15/15^b^	1/3
Behavior problems	2/3	4/5	2/9^b^	1/3b,c
Feeding difficulties	1/3	2/5	10/20	1/3
Seizures	0/3	2/5	8/19	1/3
CNS abnormality	0/1	2/3	7/20^d^	1/1
Hypotonia	0/3	3/5	5/19	0/3
Hypertonia	1/3	1/5	2/19	0/3
Eye abnormalities	0/3	1/5	8/20	1/3
**Growth**				
IUGR/small size at birth	0/2	2/4	13/16^e^	0/2
Postnatal growth retardation	0/3	4/5	15/17^b^	1/3
**Dysmorphic features**				
Thin/sparse hair	0/3	5/5	3/19	1/3
Prominent/high forehead	1/3	2/5	4/19	0/3
Dysplastic/low-set ears	0/3	4/5	16/19	1/3
Prominent nasal bridge	1/3	3/5	4/19	2/3
Macroglossia	0/3	1/5	1/19	0/3
Micrognathia	2/3	3/5	14/19	2/3
**Craniofacial**				
Microcephaly	1/3	4/5	14/20	1/3
Macrocephaly	1/3	0/5	0/20	0/3
Cleft palate	1/3	2/5	12/20	3/3
High palate	0/2	3/5	2/19	0/3
Tooth abnormalities	3/3	5/5	4/9^b^	?
**Cardiovascular**				
Heart defect	0/3	1/5	5/19	0/3
**Genitourinary**				
Inguinal hernia	0/3	3/5	2/20	0/3
Small genitalia	1/3	3/5	2/19	0/3

a Twenty patients have been reported; one did not have a physical examination [12]–[27].

b Some cases were not old enough at time of report or death to assess for this feature.

c One balanced translocation case was reported with autism spectrum disorder and developmental dyspraxia; no other information was given [9].

d Only one report specifically states lack of CNS abnormality.

e Birth data is not available in all reports.

Subject 2 is a 21-year-old male with severe mental retardation, dysmorphic features, cleft palate, and behavior problems. The subject was born to an 18-year-old G3P0→1020 mother at term via spontaneous vaginal vertex delivery following a pregnancy complicated by maternal smoking (1/2 pack per day), maternal x-rays following a car accident, and bleeding at 6 months, followed by bed rest. Birth weight was at the 50^th^ percentile. The subject had a cleft palate, which was repaired before 1 year of age. He had feeding difficulties as an infant with frequent vomiting. Developmentally, he smiled at 1 month, rolled over at 4 months, and walked alone at 2–3 years. At age 21 he does not put words together and is not toilet trained. Formal evaluation at 10 years of age put his development at 18–24 months. Dysmorphic features include a broad nasal midsection, short tongue, short oropharynx with poor movement, small mandible with crowded teeth, small testes, slightly broad thumbs and first toes, prominent heels, and macrocephaly. Figures 2C and D show subject 2's facial features at age 10. At age 21 height is at the 10^th^ percentile, weight is at the 5^th^–10^th^ percentile, and head circumference is greater than the 98^th^ percentile (59 cm). Neurological examination revealed increased tone, normal strength, and deep tendon reflexes measuring 3+. The subject has a happy demeanor, although he has a history of aggression at times and will pick his skin. Food is locked up because of overeating and vomiting, and he will eat non-food items. He also has a high tolerance for pain.

Subject 2's family history is significant for a full brother with speech delays as a child, but he is not having any problems at age 20. A paternal half-sister has a cleft lip/palate and mental retardation. The subject's father is of normal intellect. There is also a paternal first cousin who was born with a cleft palate and an unspecified ear anomaly that required plastic surgery. Two maternal half-sisters are developmentally normal. Learning disabilities were present in a maternal uncle and a maternal first cousin. Ancestry is African-American, and consanguinity was denied.

Subject 3 is a 6-year-old female with severe mental retardation. She is of Guatemalan ancestry, and no family history information is available. Developmental delays were noted at 6 months of age. At 4.5 years, developmental assessment placed her at an 18-month level. Intelligence testing showed an IQ of 32. On the Vineland Adaptive Behavior Scale, she had a standard score for adaptive behavior of 56 with strength in socialization skills and a standard score of 66 with other scores clustering in the mid 50s. She does not have any speech, but she does have some signs. The only dysmorphic features present are borderline microcephaly with some brachycephaly and fused mandibular central incisors. She does not have a cleft palate. At age 6 height is at the 75^th^ percentile, weight is at the 50^th^–75^th^ percentile, and head circumference is at the 2^nd^ percentile (48 cm). The only troublesome behavior noted by her parents was her inability to sleep through the night until she was 5 years old.

## Discussion

We report the characterization of three individuals with microdeletions of 2q33.1. The microdeletions range in size from 173.1 kb to 185.3 kb, and each spans part of *SATB2*. To our knowledge, these are the smallest deletions associated with the 2q32q33 microdeletion syndrome; the minimal deletion region in the four individuals reported by Van Buggenhout et al. [11] was 8.1 Mb. The three individuals in the present study had clinical features characteristic of 2q33.1 microdeletion syndrome, including severe developmental delay with little to no speech and tooth abnormalities (Table 1). Two of the subjects also have unusual behavior.

All three individuals in our study had dentofacial abnormalities. Subjects 1 and 2 had micrognathia or a small mandible, likely contributing to the crowding of the teeth. Several of the individuals previously reported with cytogenetically visible deletions of 2q32q33 have had dental abnormalities, including missing teeth, abnormally shaped teeth, malocclusion, and diastema [11], [14], [22], [23], [26], [28]. The role of *SATB2* in tooth and jaw development is supported by the identification of a *de novo SATB2* mutation in a male with profound mental retardation and jaw and tooth abnormalities [7] and a translocation interrupting *SATB2* in an individual with Robin sequence [8]. In addition, mouse models have demonstrated haploinsufficiency of *Satb2* results in craniofacial defects that phenocopy those caused by 2q32q33 deletion in humans; moreover, full functional loss of *Satb2* amplifies these defects. *Satb2* ^+/−^ mice have small mouths, premaxillary and nasocapsular hypoplasia, micrognathia, and variable incisor agenesis. *Satb2* ^−/−^ mice have fully penetrant incisor adontia. Loss of *Satb2* correlates with increased cell death in the developing jaw along with altered expression of other craniofacial developmental genes [10]. The individuals reported by Van Buggenhout et al. [11] also presented with thin and sparse hair and thin and transparent skin, and two had nail abnormalities. This combination of features is suggestive of an ectodermal dysplasia, although the individuals in the present study did not have any of these other ectodermal-related features. The detection of a deletion of 2q31.2q32.3 that did not encompass *SATB2* in an individual with abnormal teeth and thin skin suggests other genes in the region may impact ectodermal development [29]. Thus, *SATB2* haploinsufficiency likely contributes to the tooth abnormalities in the 2q32q33 microdeletion subjects, but deletion of other genes may be necessary for the full ectodermal phenotype.

Although haploinsufficiency of *SATB2* has been suggested to cause isolated cleft palate in humans, within our cohort *SATB2* deletions show reduced penetrance for cleft palate (Table 1). Subject 2 is the only individual in this study with a cleft palate, and his family history was significant for clefting, suggesting there could be other genetic factors contributing to the clefting. These relatives were not available for testing to determine their *SATB2* genotype. Incomplete penetrance has been observed in mouse models; one study of *Satb2*-haploinsufficient mice showed cleft palate in approximately 25% [10], whereas another population was reported as phenotypically normal [4]. Thus, *SATB2* haploinsufficiency alone may not be sufficient for cleft palate; other factors, environmental or genetic, may need to be present to lead to clefting.

*SATB2* also likely influences brain development, as illustrated by the severe mental retardation seen in these subjects. This is consistent with mouse studies that show Satb2 is necessary for proper establishment of cortical neuron connections across the corpus callosum [3], despite the apparently normal corpus callosum in heterozygous knockout mice. One subject in this report who had brain imaging, subject 1, had a normal study.

A behavioral phenotype in some individuals with 2q32q33 microdeletions further implicates a role of this gene in brain development. Subjects 1 and 2 in the present study had characteristic behaviors, including repetitive behaviors, touch avoidance, chewing on hands, skin picking, aggression, and pica. Subject 3, who did not have a history of behavior problems, did have a history of sleeping difficulties. Some of these behaviors overlap with those seen in previously reported individuals [11], [25], [26], [28]. Additionally, one individual with a balanced translocation disrupting *SATB2* had autism spectrum disorder [9]. By excluding the deletion of the individual in their study without behavioral problems, Van Buggenhout et al. [11] proposed a 0.5-Mb common deletion region at 2q32.3 proximal to *SATB2* for behavioral problems. However, the presence of a rather typical behavioral phenotype in two of the individuals in our report suggests a role for *SATB2* haploinsufficiency in these characteristic behaviors. However, not all individuals with *SATB2* abnormalities exhibit behavioral problems; the individuals reported by Brewer et al. [6] with balanced translocations disrupting *SATB2* [5] had mild learning disabilities with speech delay and no report of behavior problems. Haploinsufficiency for other genes in the region may also have an impact on behavior, because the individual with a 2q31.2q32.3 deletion that did not include *SATB2* also had hyperactivity, anxiety, aggressiveness, and self mutilation [29].

Our results suggest haploinsufficiency of *SATB2* causes some of the clinical features associated with the 2q32q33 microdeletion syndrome, including palate abnormalities, tooth anomalies, MR and behavior problems. Because many chromosomal syndromes are caused by more than one gene (contiguous gene syndromes), identification and characterization of additional individuals may elucidate the full etiology of this syndrome.

## Materials and Methods

### Ethics statement

For the individuals with 2q33.1 microdeletions described here, informed consent was obtained to perform high-resolution microarray-based molecular cytogenetic testing and to publish photographs.

### Subjects and controls

During the period encompassing November 2007 through October 2008, we tested 9,773 samples submitted to Signature Genomic Laboratories from the United States and abroad. The most common clinical presentations were mental retardation, developmental delay, or multiple congenital anomalies.

### BAC Microarray analysis

Microarray-based comparative genomic hybridization (aCGH) was performed with a BAC microarray (the SignatureChip; Signature Genomic Laboratories, Spokane, WA) [30]. The SignatureChip Whole Genome (SignatureChipWG) is currently in use in this laboratory. Results were visualized using Signature Genomic Laboratories' laboratory-developed computer software program Genoglyphix (http://www.signaturegenomics.com/genoglyphix.html). Each BAC clone was verified by fluorescence *in situ* hybridization (FISH) for its chromosomal location prior to microarray construction and validated for use in FISH to visualize chromosome abnormalities identified by the microarray. Microarray analysis was performed as previously described [31].

### Oligonucleotide Microarray analysis

Oligonucleotide-based microarray analysis was performed on individuals with microdeletions of 2q33.1 using a 105K-feature whole-genome microarray (SignatureChip Oligo Solution, made for Signature Genomic Laboratories by Agilent Technologies, Santa Clara, CA) with one probe every 10 kb in targeted regions—microdeletion/microduplication syndromes, the pericentromeric regions, subtelomeres and genes in important developmental pathways—and an average probe spacing of one probe every 35 kb throughout the rest of the genome. Genomic DNA was extracted from peripheral blood using a Qiagen M48 Biorobot automated DNA extraction system. Purified genomic DNA was then sonicated and labeled with Alexaflour dyes 555 or 647 using a BioPrime Total DNA labeling kit (Invitrogen Corp, Carlsbad, CA). Array hybridization and washing was performed as specified by the manufacturer (Agilent Technologies). Arrays were scanned using an Axon 4000B scanner (Molecular Devices) and analyzed using Agilent Feature Extraction software v9.5.1 and Agilent CGH Analytics software v3.5.14. Results were then displayed using custom oligonucleotide aCGH analysis software (Oligoglyphix; Signature Genomic Laboratories).

### Genomic sequence analysis

Computational analysis of 2q32q33 was performed using the annotated May 2006 assembly of the human genome on the UCSC genome browser (http://genome.ucsc.edu; build hg18).

### Fluorescence in situ hybridization

Deletions of the *SATB2* locus were confirmed and visualized by metaphase FISH using BAC clone RP11-404F23 as previously described [32].
